# Synchronizing immunity and angiogenesis in lung adenocarcinoma: bridging biological synergy and clinical translation

**DOI:** 10.1080/15384047.2026.2728337

**Published:** 2026-09-08

**Authors:** Pınar Peker

**Affiliations:** a Department of Medical Oncology, Adana City Training and Research Hospital, Adana, Türkiye

**Keywords:** Lung adenocarcinoma, lenvatinib, radiotherapy, angiogenesis, biomarkers

## Abstract

The recent study by Liu et al. provided compelling preclinical evidence that lenvatinib enhances the efficacy of combined radiotherapy and PD-L1 blockade by modulating both angiogenesis and antitumor immunity in lung adenocarcinoma. In this correspondence, I discuss the translational implications of these findings and highlight several challenges that should be addressed before clinical implementation. These include the molecular heterogeneity of lung adenocarcinoma, interpatient variability in the temporal dynamics of vascular normalization and immune activation, and the limitations of relying solely on PD-L1 expression for patient selection. I further emphasize the need for biomarker-driven and adaptive clinical trial designs incorporating functional imaging, circulating biomarkers, and immune monitoring to optimize treatment timing and identify patients most likely to benefit from this therapeutic strategy. These considerations may facilitate the successful translation of promising preclinical observations into precision radioimmunotherapy for lung adenocarcinoma.

Dear Editor,

I read with immense interest the recently published article by Liu et al. entitled “Lenvatinib potentiates the antitumor efficacy of combined radiotherapy and PD-L1 blockade in lung adenocarcinoma.” It is a well-designed preclinical study that expands our understanding of the complex interactions among angiogenesis, radiotherapy, and antitumor immunity. Kudos to the authors. This study provides a strong biological rationale for combining antiangiogenic therapy with radioimmunotherapy in lung adenocarcinoma by demonstrating that lenvatinib simultaneously inhibits radiation-induced PD-L1 expression, impairs VEGF/VEGFR2 signaling, and remodels the tumor microenvironment toward an immune-permissive phenotype.^1^

The major contribution of this work is not just showing additive anti-tumor activity but highlighting that vascular normalization and immune activation should be viewed as complementary instead of being regarded as independent therapeutic processes. It fits with the increasing recognition that aberrant tumor vasculature is a key contributor to immune resistance through the restriction of lymphocyte trafficking, maintenance of hypoxia and establishment of immunosuppressive cellular populations in the TME.^2^ ^,^ ^3^ In that regard, therapeutic strategies that can simultaneously target vascular dysfunction as well as adaptive immune escape may be superior to those directed at either mechanism alone.^3^ ^,^ ^4^

However, moving these promising results from the bench to the bedside is almost certainly much more difficult than the biology would imply.

One critical factor to think about is the incredible biological heterogeneity of lung adenocarcinoma. The murine model used is a suitable system to study the mechanism but cannot replicate the full molecular heterogeneity of patients. Mutations affecting STK11, KEAP1, KRAS, TP53, or EGFR supply immunologically relevant genomic alterations that greatly impact immune responsiveness and angiogenic signaling dynamics.^5^ Thus, the extent of benefit measured in experimental models should not be extrapolated across molecularly diverse patient groups.

Time gap between vascular normalization and the immune response to radiation therapy. However, the problem goes beyond the issue of treatment scheduling. It is possible that the time required for vascular normalization, immune cell infiltration, cytokine secretion, and PD-L1 up-regulation can vary greatly between individual patients, suggesting that a standardized treatment protocol may not be suitable for all. Future clinical trials might benefit from implementing an adaptive therapy approach, which could include the use of functional imaging, biomarker levels, or immune dynamics monitoring, instead of fixed administration schedules. Adaptive therapy would include functional imaging, biomarker levels, or immune dynamics monitoring.^6^

It might well be that the greatest chance coming out of this research will be in the development of biomarkers. The future of radioimmunotherapy cannot hinge entirely on the expression of PD-L1. Rather, a biomarker strategy involving angiogenic properties, immunocell profile, hypoxia-related markers, kinetics of circulating tumor DNA, T-cell receptor repertoire, and activation of the FGFR signaling pathway will likely offer a better framework for patient selection who will respond to this therapy.^7-9^ In this way, such a precise approach might end up being even more relevant than the treatment itself.

Clinically, this type of approach looks particularly appealing for patients with locally advanced but unresectable disease, oligometastatic cases with consolidated radiotherapy, and patients showing intrinsic resistance to immune checkpoint blockade. However, a biomarker-driven clinical trial will have to show that this treatment strategy is not only beneficial in terms of increased progression-free survival but also benefits certain biological groups of patients.

In summary, Liu et al. described an elegant study that contributes considerably to the development of the underlying mechanism of radioimmunotherapy for lung adenocarcinoma. Instead of the question of whether the use of lenvatinib improves radiotherapy and blocking PD-L1, the upcoming generation of clinical research needs to answer who, where, and when would benefit from such treatment. Answering those questions in a well-designed and prospective manner using biomarkers will finally determine if the synergy shown by Liu et al. is feasible in practice.

## Data Availability

No datasets were generated or analyzed during the current study.
